# Imported Human Rabies, the Philippines and Finland, 2007

**DOI:** 10.3201/eid1608.091380

**Published:** 2010-08

**Authors:** Ruska Rimhanen-Finne, Asko Järvinen, Markku Kuusi, Beatriz P. Quiambao, Fidelino F. Malbas, Anita Huovilainen, Hannimari Kallio-Kokko, Olli Vapalahti, Petri Ruutu

**Affiliations:** National Institute for Health and Welfare, Helsinki, Finland (R. Rimhanen-Finne, M. Kuusi, P. Ruutu); Helsinki University Central Hospital, Helsinki (A. Järvinen, H. Kallio-Kokko, O. Vapalahti); Research Institute for Tropical Medicine, Manila, the Philippines (B.P. Quiambao, F.F. Malbas Jr.); Finnish Food Safety Authority, Helsinki (A. Huovilainen); University of Helsinki, Helsinki (H. Kallio-Kokko, O. Vapalahti)

**Keywords:** Rabies, human, imported, viruses, lyssavirus, zoonoses, international action, Finland, the Philippines, letter

**To the Editor:** Rabies is a fatal zoonotic infection of the central nervous system caused by viruses of the genus *Lyssavirus* (*1*). Because person-to-person transmission is rare (*2*), public health action requirements in the countries where imported human rabies is seen are limited. However, communication with persons in places of exposure to provide information that could lead to public health action may not be easy.

On August 28, 2007, a 45-year-old man from the Philippines who worked on a passenger ship used for pleasure voyages was admitted to a hospital in Helsinki, Finland, because of difficulty swallowing since August 23 (Figure). He suspected rabies because he had been bitten by a dog, allegedly owned by a neighbor in the Philippines, in June. When he called home on August 27, he found out that the suspected dog was still alive.

**Figure:**
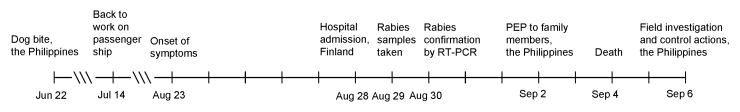
Time sequence of rabies case in a 45-year-old man from the Philippines who had been bitten by a dog, June–September 2007. RT-PCR, reverse transcription–PCR; PEP, postexposure prophylaxis.

Results of a physical examination, which included detailed neurologic tests, were unremarkable except for a subfebrile axillary temperature. Basic laboratory test results were within reference limits. Cerebrospinal fluid (CSF) contained marginally increased levels of leukocytes, erythrocytes, lactate, and proteins. Results of bacterial staining and culture of CSF were negative. Brain magnetic resonance imaging and an electroencephalogram showed no signs of encephalitis.

On August 29, the patient became febrile and disoriented, and samples (saliva, throat swab, and CSF) were obtained for rabies testing at the Finnish Food Safety Authority. A reverse transcription–PCR (RT-PCR) result for a saliva sample was positive for rabies virus RNA glycoprotein–RNA polymerase intergenic region (*3*); CSF and throat swab samples showed negative results. The virus strain (GenBank accession no. GQ856149) was closely related to genotype 1 strains isolated from dogs in the Philippines (GenBank accession nos. AB116577 and AB116578). Saliva and throat swab samples were positive by real-time RT-PCR also performed at the Institute of Tropical Medicine, Hamburg, Germany. The patient died 12 days after onset of symptoms (September 4).

The deceased patient was transported to the Philippines in an impervious body bag placed in a zinc coffin according to instructions from the Philippine consulate and the Agreement on the Transfer of Corpses (*4*). Because there are no rabies-vaccinated embalmers in Finland, embalming was not performed.

The diagnosis was conveyed to the patient’s family in the Philippines by relatives in Europe and the United States. These relatives also informed public health authorities in Finland that the dog considered to be the source of rabies was alive and had caused anxiety and accusations in the home town of the deceased. Suspected transmission from a dog still alive 7 weeks after the incident and anxiety among the population prompted a detailed investigation by the National Public Health Institute (Helsinki, Finland) and the Research Institute for Tropical Medicine (RITM; Manila, the Philippines).

On September 6, a veterinary and medical team from RITM conducted an investigation in the home town of the patient. The suspected dog, a 2-year-old mongrel, had not been vaccinated against rabies but showed no clinical signs. The family indicated that the bite in June had probably occurred in the evening, making identification of the dog difficult. With the owner’s consent, the dog was humanely killed and tested for rabies at the RITM Rabies and Special Pathogens Laboratory. The cadaver was treated with 70% formalin before it was buried. Results of a fluorescent antibody test for rabies were negative.

Relatives of the patient received rabies prophylaxis in an animal bite clinic in the Philippines on September 2 because of suspected exposure. The local government had conducted dog rabies vaccination in the area. An information campaign of lectures about rabies in the affected area was initiated, and the need for dog vaccination and stray dog control was emphasized.

An investigation of the passenger ship on which the patient worked showed that he had shared a cigarette with his cabin mate and they had drunk from the same bottle. The cabin mate was advised to receive prophylaxis. Attempts to contact the ship and shipping company in the United States were not successful. Investigation in Finland identified 33 healthcare-associated contacts before virus isolation was attempted. These persons received postexposure prophylaxis. Guidelines regarding those exposed to a rabies patient were then updated. This case highlights the need to increase awareness of rabies infection among healthcare workers.

Rapid collaboration between public health authorities in the Philippines and Finland led to appropriate action at the site of origin of the rabies case within a few days. In a country in which rabies is not endemic, diagnosing rabies and implementing control measures in healthcare settings are often difficult because of limited experience with this disease. The last human rabies case in Finland was diagnosed in 1985, when a bat researcher died after being bitten by bats abroad and in Finland (*5*). For imported cases, patient history may be incomplete, but use of RT-PCR for saliva can provide a rapid confirmation of the diagnosis. To support risk assessment and decision making, better definition of the roles of public health authorities regarding a mandate or responsibility to acquire information concerning international ships is needed.
